# Changes in Income at Macro Level Predict Sex Ratio at Birth in OECD Countries

**DOI:** 10.1371/journal.pone.0158943

**Published:** 2016-07-20

**Authors:** Ohto Kanninen, Aleksi Karhula

**Affiliations:** 1 Labour Institute for Economic Research, Helsinki, Finland; 2 Department of Social Research, University of Turku, Turku, Finland; Instituto de Higiene e Medicina Tropical, PORTUGAL

## Abstract

The human sex ratio at birth (SRB) is approximately 107 boys for every 100 girls. SRB was rising until the World War II and has been declining slightly after the 1950s in several industrial countries. Recent studies have shown that SRB varies according to exposure to disasters and socioeconomic conditions. However, it remains unknown whether changes in SRB can be explained by observable macro-level socioeconomic variables across multiple years and countries. Here we show that changes in disposable income at the macro level positively predict SRB in OECD countries. A one standard deviation increase in the change of disposable income is associated with an increase of 1.03 male births per 1000 female births. The relationship is possibly nonlinear and driven by extreme changes. The association varies from country to country being particular strong in Estonia. This is the first evidence to show that economic and social conditions are connected to SRB across countries at the macro level. This calls for further research on the effects of societal conditions on general characteristics at birth.

## 1. Introduction

The Trivers-Willard hypothesis [1] (henceforth TWH) predicts that natural selection favors a positive relationship between sex ratio at birth (SRB) and mothers’ ability to invest in offspring. This prediction stems from the assumption that a male in good condition at the end of the period of parental investment produces more offspring than a female in similar condition, and a female in bad condition produces more offspring than a male in similar condition. Although father’s active role in parental investment and kin interaction between adults complicates the application of TWH to humans, we could expect SRB to vary with environmental factors affecting condition. We do not presume this to benefit reproductive success in the current societies, but simply address the possibility that human SRB can still vary based on evolutionary conditions long gone. Indeed, sex ratio has been decreasing in several industrial countries in recent decades [2,3]. Theories as to why have been put forth, but the proposed explanations are unable to fully account for the changes [2].

Previous studies have found statistically significant associations between human SRB and multiple other factors. These studies can be roughly divided into three groups according to the explanatory variables used in them: studies concerning disasters [4–9], parental characteristics [10–16], and other country-level variables [17–21]. Notably, there are also studies showing no significant association [22–25].

Many studies give credence to TWH, but we see three caveats in the literature: the possibility of publication bias, narrow focus, and in some cases focus on the absolute levels instead of changes. The possibility of publication bias arises from the fact that almost all of the studies on human SRB study the effects of changes in conditions that have occurred multiple times throughout the history (wars, natural disasters, economic collapses). We cannot be certain, if all the similar changes result into changes in SRB, and even more importantly that not only the randomly statistically significant ones have been reported. This does not mean that the studies concerning specific disasters or events are necessarily invalid or unnecessary. It means that further research is needed to verify the results and eliminate the possibilities of publication bias.

The narrow focus to concentrate on single events is also problematic as it is not in itself enough to explain clear long-term trends in sex ratios or remaining country specific differences. This requires the analysis of long-term changes in the conditions of living.

Third caveat that we see in some of the studies in the current literature is the focus on the absolute levels not on the changes. We see that as humans are known to adapt to their environmental conditions mentally [26], it is unlikely that any sex ratio would be affected by constant levels of wellbeing, but rather the changes and shocks in it. As the living conditions have risen enormously without systematic change in sex ratio over the course of human history, we deem it unlikely that the absolute levels of wellbeing would affect sex ratios.

Our work addresses two of these three shortcomings. First, we aim to study the effects of large-scale macro-level social phenomena, not those of a specific disaster or event. This has more relevance for societies compared to narrow and specific individual-level determinants or country-level disasters. The fall in SRBs observed in multiple industrialized countries also requires an explanation that can be applied to a wide set of countries. Second, we focus on changes in disposable income–not levels. This is consistent with the idea that a sort of “hedonic adaptation” [26] renders gains of the distant past irrelevant to such sociobiological processes as SRB. As much of the literature finding significant results concerns disasters, it is likely that the shock to the status quo is the main mechanism behind the effects, and we expect humans to adapt to new macro societal conditions quite rapidly. Publication bias, however, still remains a worry.

## 2. Data and Methods

We employ OECD (2014) data for annual growth rates of real household net disposable income deflated by final consumption of households between the years 1971 and 2013 for 23 countries. We also subtract population growth from the income figures using mid-year estimates of total population from the World Bank (2015) to obtain per capita figures. We link this information with annual live birth data by sex obtained from the United Nations (2014), which is a dataset collected through questionnaires to national statistical offices since 1948.

This gives us an unbalanced panel of countries from 1971 onwards, where for each year for each country we have the change in disposable income for that year and also the SRB. The panel is unbalanced, meaning that for some countries the period covered is not as long as for others. In the analysis, we only included countries with more than 10 years of data (see S1 Table for included countries). We further excluded South Korea from the countries remaining after this selection. This was due to fear of possible sex-selective abortion and certain anomalies in the South Korean live birth data.

We employ fixed effects regression model of the following form to estimate SRB:

$$SRB_{i,t}=\alpha +\gamma_{i}+\delta_{t}+\beta \Delta Y_{i,t}+\varepsilon_{i,t},$$ (1)

where *i* and *t* stand for the country and year indices, respectively. *β* is the estimated association between SRB (*SRB*_*i*, *t*_) and change in disposable income (Δ*Y*_*i*, *t*_).*γ*_*i*_ and *δ*_*t*_ are the country and year fixed effects, respectively. Descriptive information on the variables can be found in S2 Table.

## 3. Results

**Fig 1:**
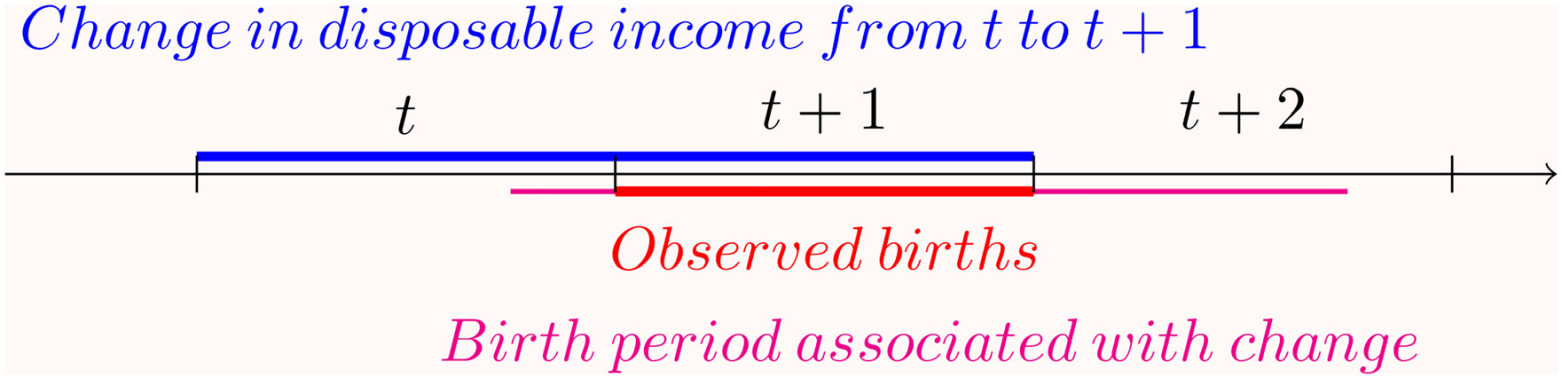
The timing of birth if conception happens at the time of income change from year t to year t+1. When examining the income change at the time of conception at the macro level from t to t+1(presented in blue), the associated birth period with conception happening during the income change is presented in purple. From this period we have taken the only full year (t+1, presented in red) that corresponds to the period of income change when calculating the SRB.

The timing of the change in disposable income and observed births is illustrated in (Fig 1). Lacking monthly data, we have associated the income growth to the interval of births mostly affected by it. We always measure the income growth taking place from one year (t) to the next (t+1) and births happening in the next year (t+1). As the income growth takes place somewhere during either year t or year t+1 the children conceived during the period are given birth during end of year t, during year t+1 or during the first three quarters of t+2. We associated the year t+1 with the income growth as this was the most likely year of birth for conceptions during the income growth period. This imperfect modeling can be expected to result in more noise in the estimates, and thus it will attenuate the observed statistical associations.

**Fig 2:**
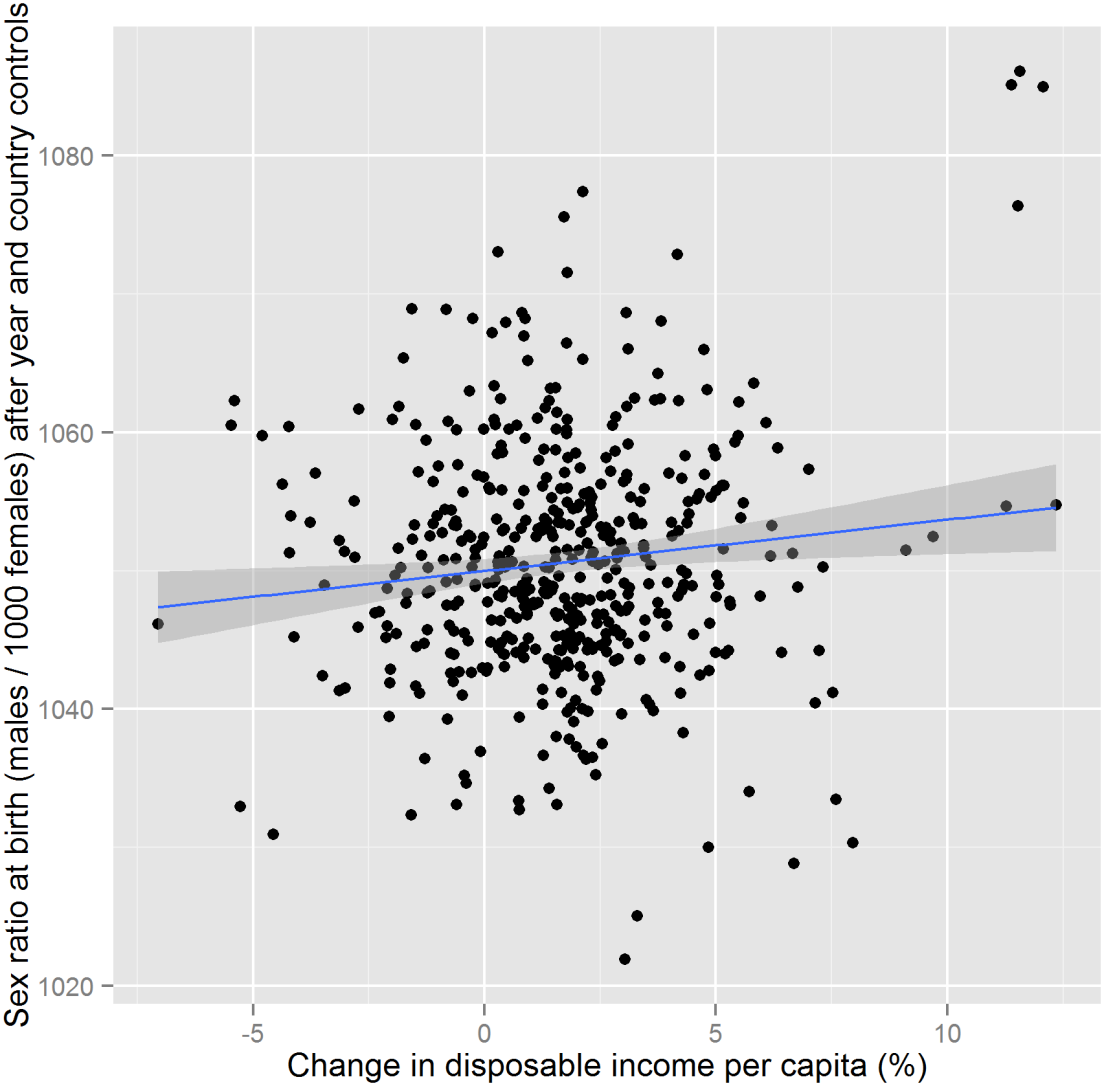
Linear association between changes in disposable income per capita from previous year and SRB net of year and country fixed effects. The x-axis of the scatter plot is the relative per capita change of real household net disposable income deflated by final consumption of households expressed in percent as calculated by the OECD between the years 1971 and 2013 for 23 countries for our main sample. The population growth numbers were obtained from the World Bank. The y-axis is SRB after controlling for year and country effects. SRB is calculated as the number of male births per 1000 female births from annual live birth data by sex obtained from the United Nations. The year and country effects are estimated with a fixed effects panel regression. The blue line represents the linear regression estimate. The dark gray area around the blue line is the 95% confidence interval.

Fig 2 shows the association between the country-level SRB and the relative percent change in disposable income at the yearly level for an unbalanced panel of 23 OECD countries for the years 1971–2012 controlled for year and country effects. The correlation between the two variables is 0.11. It is significantly positive with a p-value of 0.018. Excluding the four observations at the top-right of Fig 1 renders the estimated relationships insignificant. All four observations are for Estonia, which is almost the only country with over 10 percent increases in disposable income. Of the two other above 10 percent increases one is from Estonia and the other one from Czech Republic. The years for which Estonia experienced above 10 percent increase were 1997, 2000 and 2005–2007. EU access in 2004 most probably explains the high increases in income in mid-2000s and might have directly increased the SRB. Our sensitivity analysis also indicates that the association might be stronger in extreme cases (see S3 and S4 Figs). However, we cannot draw definite conclusions based on our current data.

To control for unobserved, time-invariant, country-level heterogeneity and common yearly variation, we introduce a fixed effects regression model to estimate SRB (see S1 and S2 Figs for the distributions of sex ratio by country and year). This simple analytical strategy leads to time- and country-independent effects of disposable income. The effect of a change in disposable income on SRB is significant at the 5% level (Table 1). A coefficient of 0.391 means that a one percent growth in disposable income is associated with an increase of 0.39 male births per 1000 female births. A one standard deviation increase in the change of disposable income is associated with an increase of 1.03 male births per 1000 female births. Including South Korea would almost triple the point estimate of the relationship.

**Table 1:** Association between changes in disposable income, GDP per capita, and changes in SRB with year and country fixed effects included in the model.

	Dependent variable: SRB
Per capita annual proportional change in disposable income, per cent	0.391** (0.157)
N	490
R^2^	0.014
Adj. R^2^	0.013

P-value of the estimate is 0.013. The explanatory variable in the fixed effects panel regression is the relative per capita change of real household net disposable income deflated by final consumption of households expressed in percent as calculated by the OECD between the years 1971 and 2013 for 23 countries for our main sample. The population growth numbers were obtained from the World Bank. The dependent variable is SRB. SRB is calculated as the number of male births per 1000 female births from annual live birth data by sex obtained from the United Nations. The year and country effects are controlled with a fixed effects panel regression.

We also show that the GDP level per capita does not have a statistically significant relationship with SRB for the countries and years studied (see S3 Table in supplementary information, columns 2 and 3). Standard of living per se is unlikely to be a significant determinant, since SRBs have decreased in the industrialized world as living standards have risen [2]. However, as the pace of change in living standards has slowed [27], SRBs have decreased as well [2].

## 4. Discussion

Our estimate shows a highly significant association between disposable income change and SRB and is thus consistent with the prediction of TWH. The estimated relationship remains even after controlling for levels. Also, consistent with the hedonic adaptation theory [26], changes in instead of levels of the standard of living exhibit a significant association. However, the result should be interpreted with caution. The estimated relationship varies strongly from country to country. In these analyses, we already exclude South Korea, which exhibits a very strong relationship. Most of the observations with large increases in disposable income remaining in the data were from Estonia. This decreases the robustness of our results. We control for country-level and yearly variation by using a fixed effects model, but change in income might simply act as a proxy for other social variables that in turn affect SRB. If those variables vary differently in countries over time, they still remain uncontrolled for. It’s unlikely that there is a causal effect from SRB to income change.

The implication of the TWH is that SRB “in mammals is a measure of tendency to invest in one sex more than in the other” since “slight advantages in condition should (because of male competition to inseminate females) have disproportionate effects on male reproductive success compared to the effects on female reproductive success” [1]. Our result suggests that TWH works also when the whole population of a country improves its condition. Besides working through straightforward mechanism of more advantaged conditions of oneself, this could work through two alternative mechanism. First, the “slight advantage” gained when the country improves its income is with respect to neighboring countries. Second, when all females improve their condition, they update their information on the condition of the other females in the country with a lag and thus erroneously perceive an advantage that actually isn’t there.

Based on the previous literature it is likely that the effect of the income growth on human SRB works through the maternal stress. Our study is not the first one to find an association between economic conditions and SRB. Further, we do not mean to imply that income is the ultimate cause for the changes. We use change in income as an explanatory variable, due to its wide availability. Income is a good measure of condition in the sense that growth income is usually associated with many positive developments such as improvements in institutions, nutrition and health [28–30]. A better measure might well be the unemployment [21] or more general political, social and economic conditions [4]. However, our result does not allow us to be precise about what facet of social development is most closely associated with SRB. We leave this for future research.

This is the first evidence of economic and social conditions influencing SRB at the macro level in a wide range of countries and underlines the significance of feedback loops between biological and social conditions. The results also offer a possible explanation to the puzzle of falling SRBs. However, it should be noted that we do not explain a large proportion of the variation in sex ratios. This could be expected especially as our data are not measured on monthly basis and are measured at country-level. Further research is needed to clarify and replicate the results in more micro-level contexts.

## Supporting Information

S1 Dataset Dataset used for the analysis. (CSV)

S1 Fig Densities for sex ratio for 23 OECD countries from 1971 to 2013. The x-axis of the scatter plot is SRB, and the y-axis presents the kernel density estimates. SRB is calculated as the number of male births per 1000 female births from annual live birth data by sex obtained from the United Nations. Only countries with more than 10 years of data are included. In addition, South Korea was omitted due to fear of possible sex-selective abortion and certain anomalies in the South Korean live birth data.
(TIFF)

S2 Fig Densities for sex ratio by year from 1971 to 2013. The x-axis of the scatter plot is SRB, and the y-axis presents the kernel density estimates. SRB is calculated as the number of male births per 1000 female births from annual live birth data by sex obtained from the United Nations. Only countries with more than 10 years of data are included. In addition, South Korea was omitted due to fear of possible sex-selective abortion and certain anomalies in the South Korean live birth data.
(TIFF)

S3 Fig Non-linear, third degree polynomial association between changes in disposable income per capita from previous year and SRB. (TIFF)

S4 Fig Non-linear, fifth degree polynomial association between changes in disposable income per capita from previous year and SRB. (TIFF)

S1 R Code R code used for the analysis. (R)

S1 Table The number of observations per country. (PDF)

S2 Table Descriptive statistics. (PDF)

S3 Table Association between changes in disposable income, GDP per capita, and changes in SRB. (PDF)
