# Supplementary figures and images for: Changes in Income at Macro Level Predict Sex Ratio at Birth in OECD Countries
Source: PLoS One. 2016 Jul 20;11(7):e0158943. doi: 10.1371/journal.pone.0158943 (PMC4954671; doi:10.1371/journal.pone.0158943)

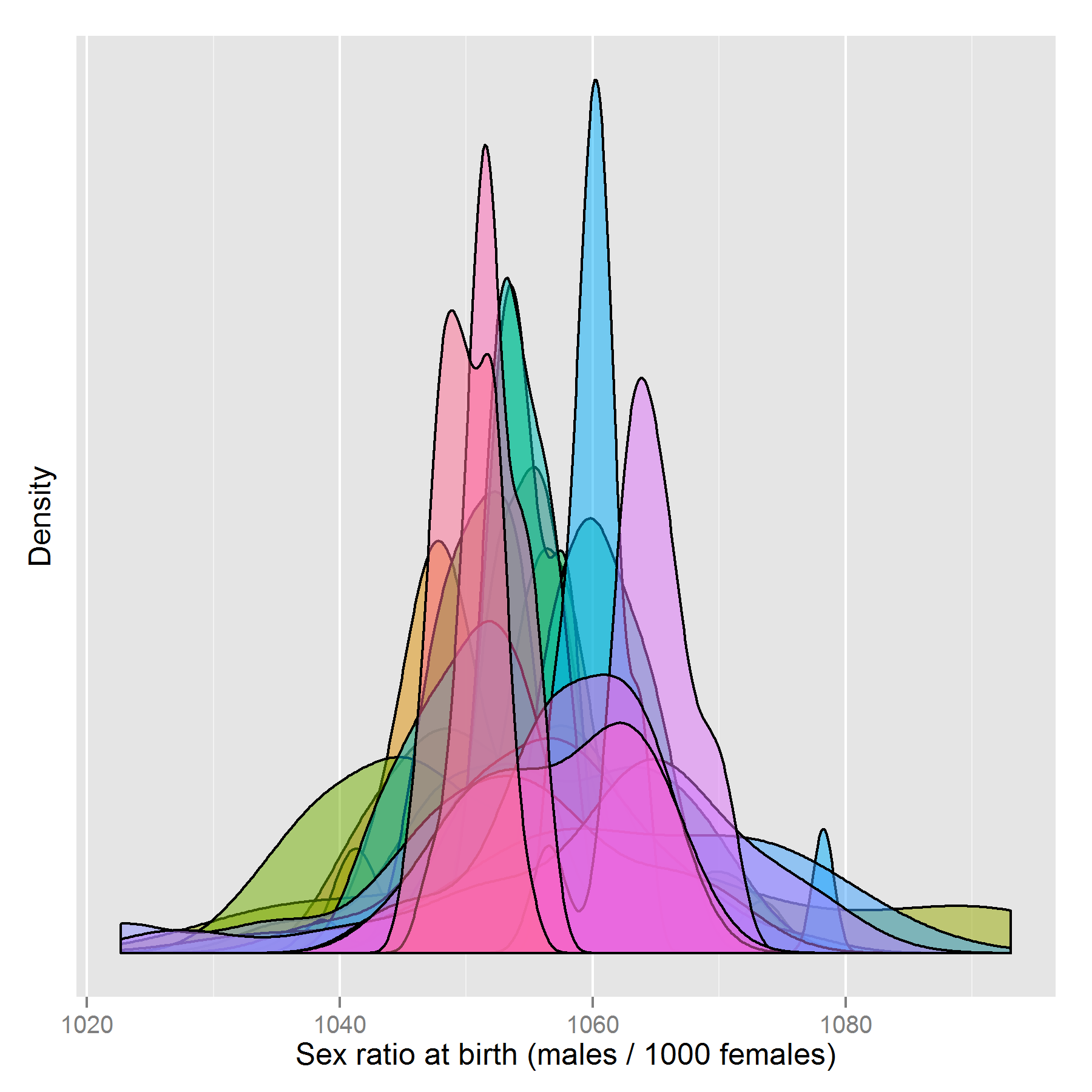

Supplement: S1 Fig — The x-axis of the scatter plot is SRB, and the y-axis presents the kernel density estimates. SRB is calculated as the number of male births per 1000 female births from annual live birth data by sex obtained from the United Nations. Only countries with more than 10 years of data are included. In addition, South Korea was omitted due to fear of possible sex-selective abortion and certain anomalies in the South Korean live birth data. (TIFF) [file pone.0158943.s002.tiff]

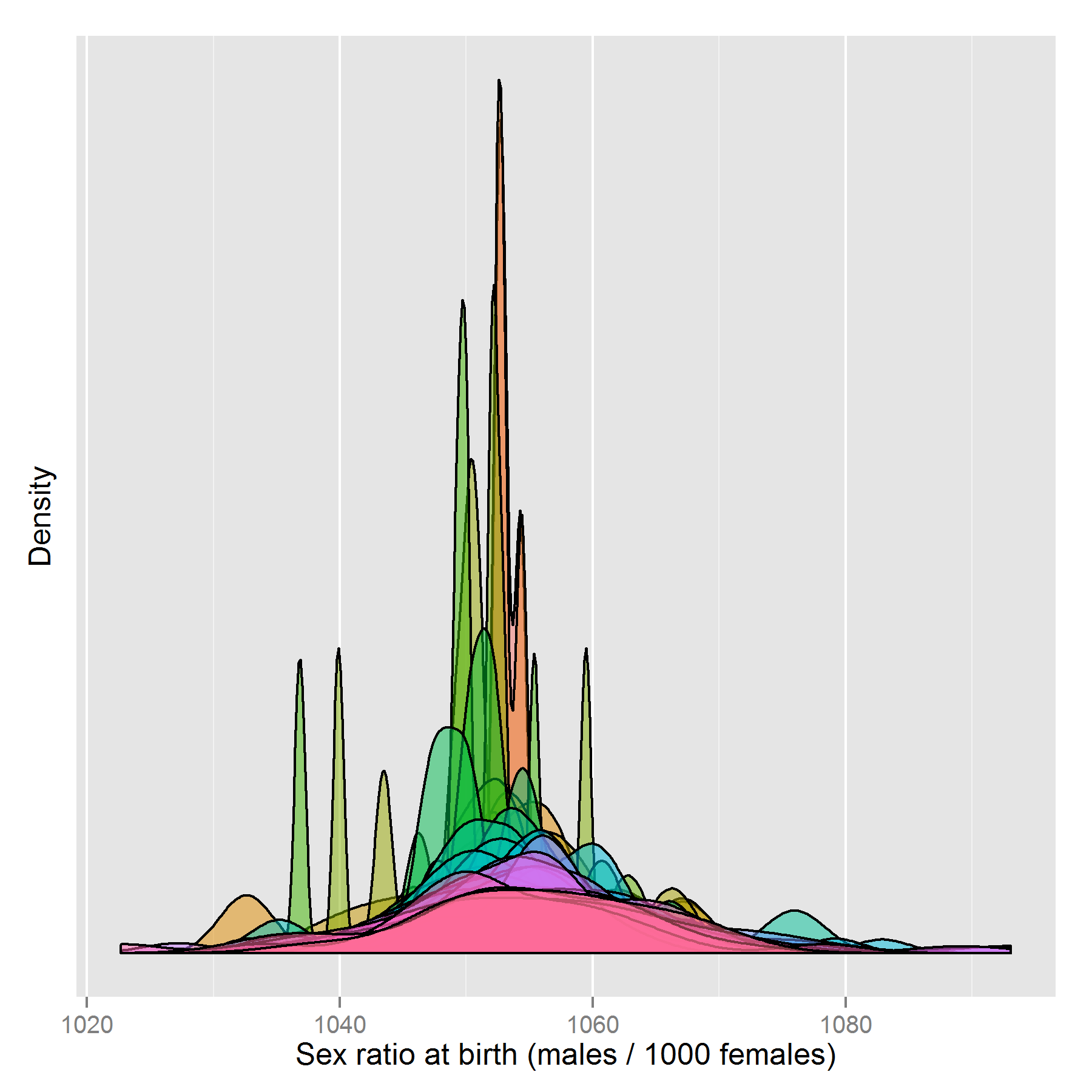

Supplement: S2 Fig — The x-axis of the scatter plot is SRB, and the y-axis presents the kernel density estimates. SRB is calculated as the number of male births per 1000 female births from annual live birth data by sex obtained from the United Nations. Only countries with more than 10 years of data are included. In addition, South Korea was omitted due to fear of possible sex-selective abortion and certain anomalies in the South Korean live birth data. (TIFF) [file pone.0158943.s003.tiff]

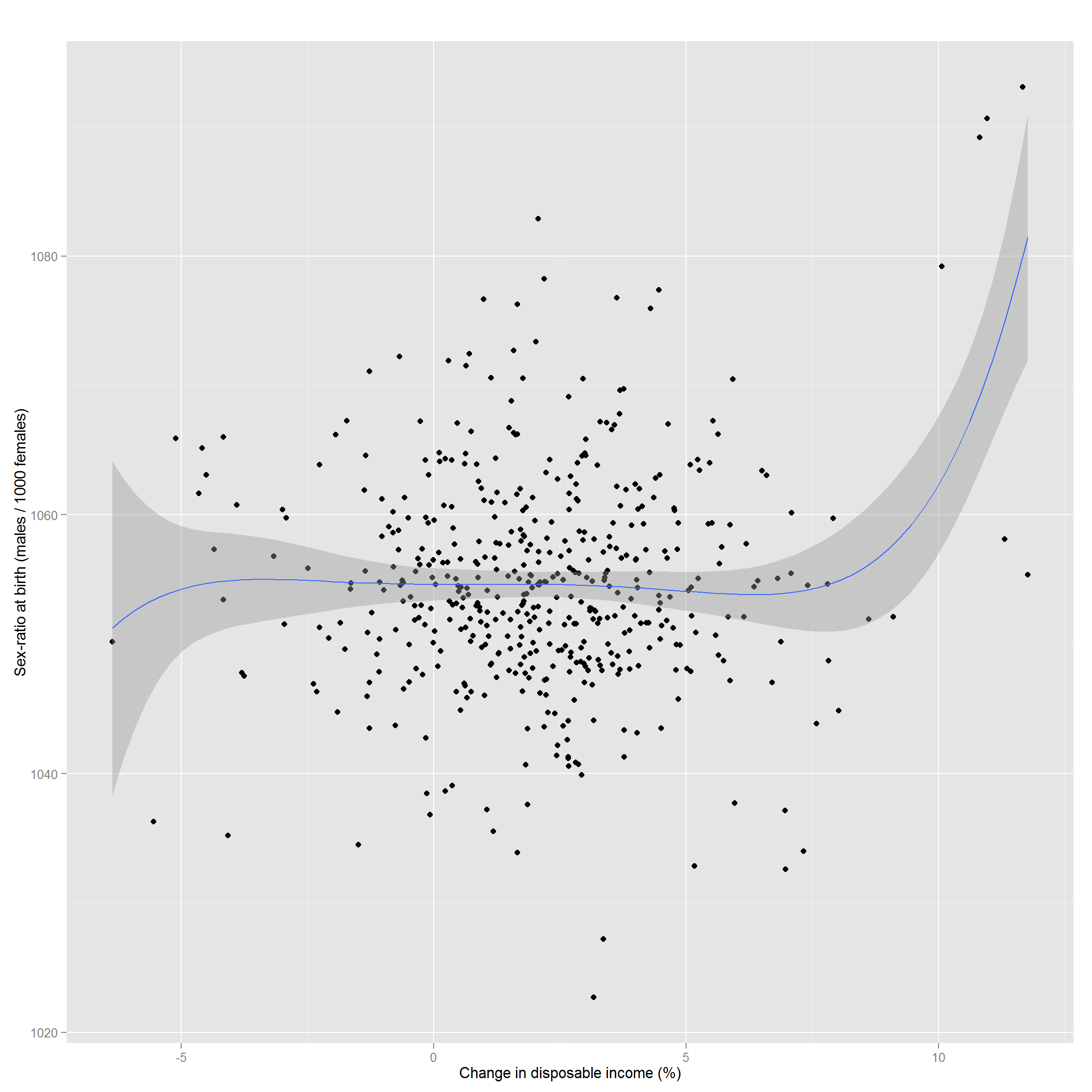

Supplement: S3 Fig — (TIFF) [file pone.0158943.s004.tiff]

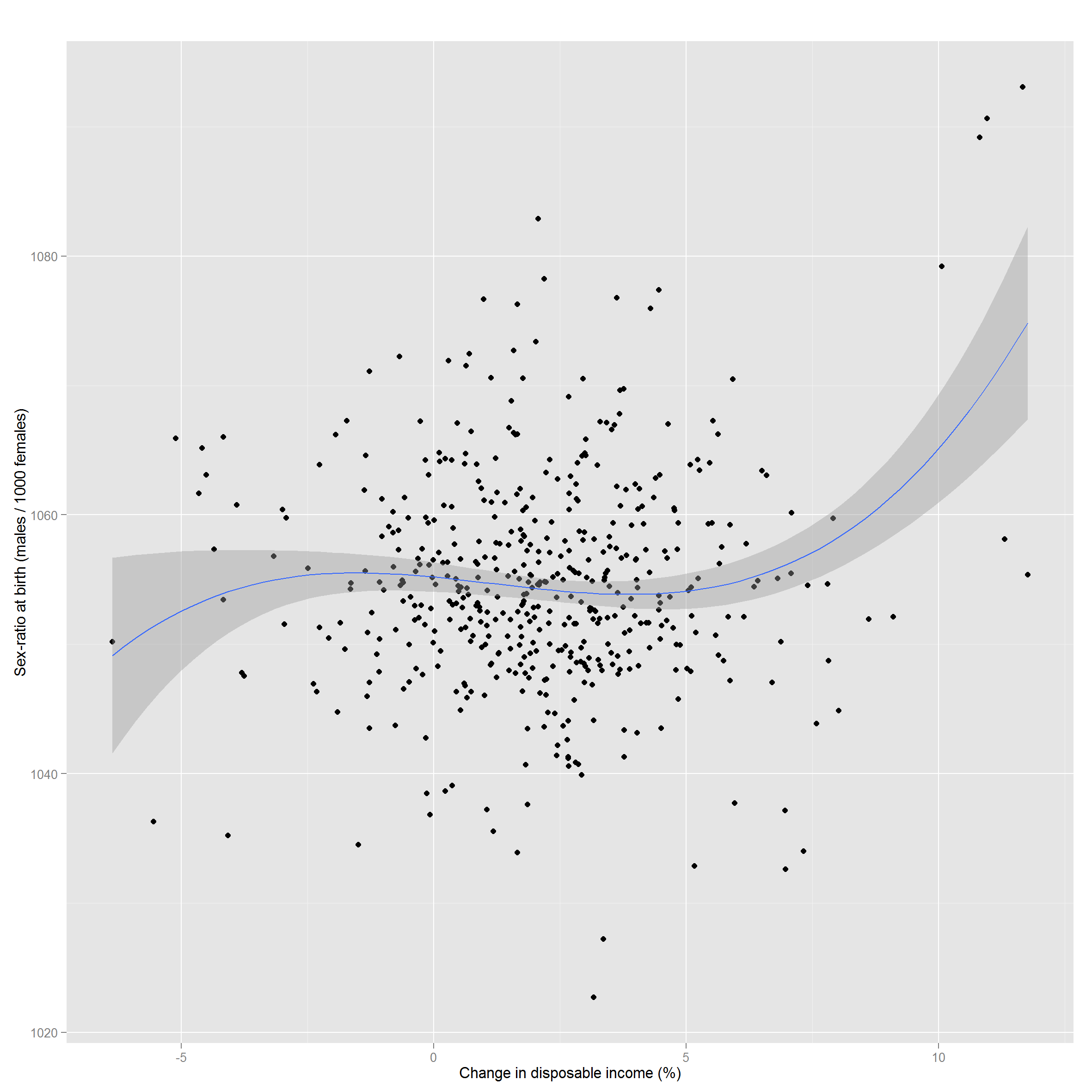

Supplement: S4 Fig — (TIFF) [file pone.0158943.s005.tiff]
