# Supplementary material for: Changes in Income at Macro Level Predict Sex Ratio at Birth in OECD Countries
Source: PLoS One. 2016 Jul 20;11(7):e0158943. doi: 10.1371/journal.pone.0158943 (PMC4954671; doi:10.1371/journal.pone.0158943)
Supplement: S1 Table — (PDF) [file pone.0158943.s007.pdf]

Table S1. The number of observations per country.

Country	Observations
Australia	41
Austria	17
Belgium	17
Czech Republic	19
Denmark	17
Estonia	17
Finland	34
France	34
Germany	17
Hungary	17
Italy	21
Japan	17
Netherlands	22
Norway	32
Poland	17
Portugal	17
Slovakia	15
Slovenia	17
Spain	12
Sweden	17
Switzerland	16
United Kingdom	21
United States	36
Total	490
