# Supplementary material for: Changes in Income at Macro Level Predict Sex Ratio at Birth in OECD Countries
Source: PLoS One. 2016 Jul 20;11(7):e0158943. doi: 10.1371/journal.pone.0158943 (PMC4954671; doi:10.1371/journal.pone.0158943)
Supplement: S2 Table — (PDF) [file pone.0158943.s008.pdf]

Table S2. Descriptive statistics.

Variable	Mean	Sd	Min	Max
Change in disp. income, %, per capita	1.7	2.6	-7	12.4
SRB	1054.8	8.6	1022.7	1093.1
GDP per capita, thousand dollars	27.8	8.2	8.4	49.1
