# Supplementary material for: Changes in Income at Macro Level Predict Sex Ratio at Birth in OECD Countries
Source: PLoS One. 2016 Jul 20;11(7):e0158943. doi: 10.1371/journal.pone.0158943 (PMC4954671; doi:10.1371/journal.pone.0158943)
Supplement: S3 Table — (PDF) [file pone.0158943.s009.pdf]

Table S4. Association between changes in disposable income, GDP per capita, and changes in SRB.

	Dependent variable: SRB		
Per capita annual proportional change in disposable income, per cent	0.391** (0.157)		0.389** (0.158)
GDP per capita, thousand		0.104 (0.256)	0.045 (0.255)
Year fixed effects	Yes	Yes	Yes
Country fixed effects	Yes	Yes	Yes
N	490	490	490
R^2	0.014	0.0004	0.015
Adj. R^2	0.013	0.0003	0.013

*Note: *p<0.1; **p<0.05; ***p<0.01*
